# Bridging disciplines-key to success when implementing planetary health in medical training curricula

**DOI:** 10.3389/fpubh.2024.1454729

**Published:** 2024-08-06

**Authors:** Ebba Malmqvist, Anna Oudin

**Affiliations:** ^1^Division of Occupational and Environmental Medicine, Lund University, Lund, Sweden; ^2^Division of Sustainable Health, Umeå University, Umeå, Sweden

**Keywords:** planetary health, one health, medical training, medical curriculum, climate change

## Abstract

Planetary health is being or should be added to medical training curricula in accordance with association consensus. Several articles published in recent years have addressed concern on the implementation, and the challenges that can occur if not addressed properly. This scoping narrative literature review focuses on planetary health as a concept, as well as challenges and suggested solutions to address these challenges. Planetary health is an important concept and needs to be addressed in all medical training. We found that one main challenge is implementation without ensuring the right competences and resources. Medically trained teachers set out to understand and teach complex natural and social systems. At some institutions the time allocated to teach planetary health is limited or non-existent. Case studies and student led teaching are solutions suggested, while other argue that true interdisciplinarity by inviting experts are more in line with what we expect from other subjects. In conclusion, the roots of planetary health, the enormous health risks at stake and nature of the subject requires medical training to adopt a true inter/trans-disciplinary approach to succeed. It might not be expected for all students to become planetary health experts, but all need a general understanding of the most important aspects and values.

## 1 Introduction

Planetary health is increasingly added to the medical training curricula in accordance with the international medical training association consensus. This is very promising, but several articles have raised challenges if the topic is not addressed properly.

### 1.1 What is the planetary health concept and where did it come from?

This little blue planet, perfectly formed for human life, has been our home for thousands of years. Earth systems enabled human life under the best conditions, a state that lasted for around 10,000 years, referred to as Geological period “Holocene.” The anthropogenic (human-made) impacts on the planet’s natural systems have led to civilizational successes of the past decades (e.g., increasing life expectancy and reducing poverty) coupled with negative global developments (e.g., loss of biodiversity, pollution and climate change). These trends are in an historical perspective quite new, and often coupled with industrialization toward the end of the 19th century with an increase particularly from the 1950s onwards (1).

The alarm bell started in the early 1960s with Rachel Carson’s book Silent Spring on man-made chemicals leading to a decline in birds (2). In 1972, Dr. Sargent wrote about the connection between the ‘planetary life-support systems’ and health and well-being (3). At about the same time, Gennady Tsaregorodtsev called for a new integrative hub of science called ‘planetary public health’ to better understand the evidence on the human health impact of environmental degradation (4). In 1980, Friends of the Earth expanded the World Health Organization definition of health, stating: “*health is a state of complete physical, mental, social and ecological well-being and not merely the absence of disease – personal health involves planetary health*” (5).

Some scholars highlighted that the idea that planetary health wasn’t a new concept; rather, these ideas have been deeply embedded within Indigenous cultures for centuries (6). The first Navajo woman surgeon said “*human health is dependent upon planetary health and everything must exist in a delicate web of balanced relationships*” (7). We should thus be humble when we present planetary health as a novelty topic or ‘new discipline’.

Planetary health can be seen as a concept that affects all healthcare providers and understood by our ancestors. This relates to indigenous knowledge, but also western ancient roots had a medical interest in the environment. The Hippocratic text *On Airs, Waters, Places* advised physicians to attend to all aspects of the environment. It took some decades for the planetary health term to become a term in mainstream modern medicine since its reintroduction in the 1980’s. The success and widespread notion of the concept came with the highly-cited 2015 keystone report by the Rockefeller-Lancet Commission on Planetary Health (1). The report defined planetary health as “*the health of human civilization and the state of the natural systems on which it depends*,” with its stated goal to find ‘*solutions to health risks posed by our poor stewardship of our planet*’ (1), the term planetary health (and what it represents) had finally entered the lexicon of mainstream medicine. The *Commission* report calls for the application of interdisciplinary knowledge, as well as input from healthcare professionals to play important its roles by supporting environmental and social sustainability (1).

### 1.2 Planetary health in medical training curriculum

Medical schools are now being called to develop physicians with the skills necessary to navigate the planetary health crisis, including the natural science and policy transformation necessary. It has been argued that a physician’s place is at the individual patient’s bedside, but now also to preventatively advocating for public health beyond the bedside. Numerous medical societies and organizations have called climate change the single greatest threat to human health (8, 9). Medical students have been pushing for integration of climate health content in curricula to equip them to adequately care for patients in a rapidly changing environment (10).

Still the medical schools have failed to adapt fast enough. The International Federation of Medical Students’ Associations conducted a survey in 2019 and 2020 in 2817 medical schools in 112 countries. Only 15% of medical schools had incorporated climate change and health and only 11% added air pollution and health into the curriculum (11). Another study covering 45% of the UK medical schools found a large disparity in the education that medical students receive on planetary health and sustainability topics, with many schools not prioritizing the field. The extent of teaching varied considerably among courses with a mean estimate teaching time of only just over two hours (12). Another study found that faculty often lack the knowledge to teach this emerging subject (13). In a US study using in-depth interviews found personal expertise as a barrier in applying climate change aspects in their teaching material or knowledge often bound to a specific person (14).

### 1.3 Aim

This narrative review article aims to give the reader a deeper understanding of planetary health as a concept and to explore challenges and solutions related to the interdisciplinary aspects of planetary health in medical curricula.

## 2 Method

This mini review used a narrative review approach which is considered appropriate when in relation to a collection of quantitative studies that have used diverse methodologies or theoretical frameworks. Narrative reviews are a useful way of linking together studies on different topics related to a new concept or to understand the historical perspective of a new concept (15). We conducted a search on PubMed on the term `planetary health’ and ´education´, ´medical curricula´ or `interdisciplinary` and ´transdisciplinary´. We also expanded the references in the articles we had found by so-called citation searching, and snowball searching. As it is a narrative mini-review we had to make limitations if similar statements had already been included.

## 3 Results

### 3.1 Inter/transdisciplinary approach vital to planetary health

In the result section we will first highlight key finding on importance of inter/trans/multi-disciplinary in medical training of planetary health. In the next chapters we will take a deeper look into some disciplines that have been addressed in the literature.

Several frameworks to address planetary health into medical curricula has been suggested (16). This includes the Planetary Health Report Card, a student developed metric tool for evaluating and improving the planetary health content (17). Sustainable Healthcare Education network has developed methods of including planetary health literacy in clinical training, such as deeper understanding on how the environment is degraded, how this impact our health and what actions can lead to improvement (18). Along with learning the science behind environmental health, students need to develop skills to lead and advocate for community change according to the Association for Medical Education in Europe consensus (19). One suggested way to get students involved is working with case studies (20). Building students’ commitment to planetary health approaches requires engaging students in interdisciplinary active learning of a transformative systems-based paradigm (21). Other concepts suggest expanding interdisciplinarity to indigenous reciprocal stewardship of our natural surroundings (22, 23). Planetary Health Education Framework also highlights the importance of transdisciplinary knowledge (including epistemological diversity) (24). Climate Crisis and Clinical Practice initiative also highlights the need for medical training to include a multidisciplinary approach (9).

The One Health and Planetary Health approaches are increasingly influencing the field of medicine way of thinking in everyday clinical practice and research (25). Both approaches represent the integrative consideration of health topics against the background of other sciences. Particularly characteristic of planetary health are, among other things, the aspects of a transdisciplinary approach (26), and this was also the findings of the methods/frameworks we identified above. Another narrative review also had similar results of the need to work interdisciplinary when including planetary health in medical training. They also highlighted the importance to work across sectors to reach a better understanding of the interactions between humans and its surrounding environment (27). Based on the seemingly importance of inter/trans-interdisciplinary curricula we will highlight some important disciplines we found in the literature and how they could be addressed. This should not be seen a complete list of an ever-expanding field.

#### 3.1.1 Natural sciences

With global health burdens shifting from infectious to non-communicable diseases (NCDs), we need greater emphasis on the health-mediating role of lifestyle and the human-manufactured threats to life within the biosphere (28). Irreversible changes to our environment have already occurred that are affecting the health of the world’s population, also known as triple crisis. Environmental pollution can be detected in the most remote areas of the earth (29), and the consequences of climate change are measurable and visible (30). The natural areas are diminishing at high speed caused by anthropogenic (human made) environmental changes to the land (31).

The concept of Planetary health in the Rockefeller report was influenced by (32, 33) models of planetary stress limits. The boundaries represent components of Earth system critically affected by anthropogenic activities and relevant to Earth’s overall state. For each of the boundaries, control variables are chosen to capture the most important anthropogenic influence at the planetary level of the boundary in focus. So-called tipping points were quantified, the exceedance of which results in the relatively abrupt and irreversible changes for the Earth system. These changes can challenge the socio-ecological resilience of societies and be catastrophic for societies and individuals alike (32).

For planetary health action to happen Anthony McMichael, one of the first epidemiologists to study the links between climate change and human health says: “*The health sector*,” McMichael demanded, “*must lift its gaze to bigger, ecological horizons. This will require […] an ability to collaborate with unfamiliar disciplines*” (34).

The future leaders in medicine need to understand the basis of our natural life-supporting systems and their boundaries. This includes a profound knowledge of natural laws on which our life support systems depend (22, 23).

#### 3.1.2 Political and economy sciences

A central characteristic of planetary health are also the terms of the urgency of transformative measures (35). It was physicians and the nascent public health movement of the 19th century that demanded the reforms in urban sanitation (36), workplace hazards (37), and battled the tobacco industry and often indifferent governments for tobacco controls (38). But never have the stakes been higher, or the scale greater, than what ecological crises now entail. The survival of our societies as we know them depends on medically informed political responses to the disruption of our planet’s human life–support systems. This will require augmented skills in health promotion principles, and deeper knowledge for health professionals to politically mobilize through social, economic and environmental advocacy for urgent and major reforms (39). The World Health Organization ask for health actors that can identify and accelerate those climate change mitigation actions that brings the greatest health gains, including the promotion of healthy urban transport systems and diets (40).

The Association for Medical Education in Europe (AMEE) has made a consensus statement *Planetary health and education for sustainable healthcare* intended to inform national and global accreditation standards, planning and action at the institutional level as well as the role of individuals in transforming health professions education. They state: “*health professions education must equip undergraduates, and those already qualified, with the knowledge, skills, values, competence, and confidence they need to sustainably promote the health, human rights, and well-being of current and future generations, while protecting the health of the planet.”* As an example they mention the skills to model co-benefits to people and planet of socio-ecological informed health programs (19). Changes that are implemented in the spirit of climate protection usually contain an additional benefit (co-benefit) for health and vice versa as exemplified by environmentally friendly forms of transport and other lifestyle factors (41). Skills to understand these co-benefits and to quantify them also in economic terms puts planetary health on par with other agendas in political and economic discussions.

#### 3.1.3 Social sciences

The consequences of human interaction with the earth’s natural systems are diverse, interconnected, and global because of globalization and the sheer scale of human resource use and consumption. The fact that impact can be both local and global, and often unevenly distributed, makes environmental justice central in planetary health. Rich nations in the global north are primarily responsible for the transgression of planetary boundaries, such as causing significantly higher CO_2_ emissions. But the effects of which are felt most acutely by poorer countries in the global south (30). Social determinants of health can either improve or exacerbate vulnerability to poor health outcomes associated with climate change, pollution, and access to green areas. Knowledge of vulnerable groups by age, culture and socio-economy and other determinants are important to consider when setting health recommendations (42). This makes it important to get a deeper understanding of inequality and justice perspectives of planetary health.

#### 3.1.4 Medical science

Resident physicians need to be equipped to care for patients affected by climate-mediated disease and advocate for solutions to the climate crisis. One approach is to organize evidence-based topics in climate and health by medical subspecialty and integrated them into pre-existing lectures in the longitudinal, outpatient lecture series (43). This will still require that students have some background knowledge on ecosystem services and planetary boundaries. Humans are inter-linked with the necessary life-support systems of this planet. In total about one quarter of all global preventable premature death is due to environmental risk factors (44).

Future physicians must be aware of, for example changes in infectious disease patterns due to lack of clean air and changing weather patterns affected by flooding and temperatures. The planetary boundary *novel entities* relate to released unsafe chemicals which are directly linked to health. Novel entities can be exemplified by the release of Per-and polyfluoroalkyl substances (PFAS) which can be present in municipal drinking water and reduce antibody levels in response to vaccination (45). Another planetary boundary is the aerosols which relates to air pollution of combustion particles that can penetrate the alveolar blood-gas exchange and causes about 7 million premature deaths a year (44). The planetary boundary, nitrogen excess, impacts ecosystems by eutrophication and more directly humans by drinking water quality. The health impacts is early in life the blue baby syndrome and later in life an increased risk of cancer (46). Planetary health is also not only about global changes but to understand local exposures. Future physicians need to develop a system thinking of these complex interrelationships with patients in their entire environment and how social determinants can impact health effects.

## 4 Discussion

Gaining support from medical school faculty can be a major challenge. In a study of eleven medical schools in UK, one educator said that sustainable healthcare ‘*was at first seen as one of my pet extra projects*’ (47). It was suggested that with time as more residency programs incorporate environmental-related content into their curricula, faculty will become more familiar with these important topics and allocate time and resources (43). Moreover, an overarching paradigm of higher education often upholds ideologies of individualism and meritocracy and a shift toward skills in compassion-knowledge-reflection are highly needed (22). The need of planetary stewards was put forward by 126 Nobel prize laureates in their 2021 statement *Our planet, our future*. One suggested way of getting an increased understanding of our interlinkage with our planet and thus the importance of biodiversity is getting the subject near the heart of the students. One example is the understanding of biomimetics for curing diseases as one third of medications used in healthcare originate from nature, the development of future cures depends on preserved diversity (48). For others it can be beneficial with the introduction of the ecosystem framework which categorize the benefits of healthy functioning ecosystems that regulate (climate), support (water, food, medicine, air) and provide services for human health and wellbeing (49). Even though this concept still has an aspect of anthropocentric (human-centered) thinking seeing nature purely as goods, it can be a steppingstone for those furthest from a more eco-centric planetary health thinking.

Teaching planetary health to students presents unique challenges, especially when some students may deny the human impact on climate change. This mirrors societal skepticism, where vocal climate change deniers exist despite overwhelming scientific consensus. It is crucial for lecturers to be exceptionally knowledgeable, capable of engaging in informed discussions with students who question the human role in climate change and planetary health. Most students have on the other hand grown up aware of insufficient actions against climate change, mass species extinctions, and pervasive pollution. This background can lead to feelings of hopelessness for some students, making the topic particularly heavy during their early clinical training years. Therefore, it is imperative that educators in planetary health maintain a high level of expertise. They must be equipped to address the skepticism of some students while also supporting those who may feel overwhelmed by the gravity of environmental issues (50). Balancing these perspectives with scientific rigor and empathy is essential for fostering a constructive and educational environment.

Using only medically trained faculty is deemed to fail in teaching a subject such as planetary health. Management of “wicked problems,” messy real-world problems that defy resolution, requires thinkers who can transcend disciplinary boundaries, work collaboratively, and handle complexity and obstacles (51). Medical training would benefit from including faculty researchers from a range of disciplines across the natural, social and health sciences (52, 53). An in-depth study of one medical school on successes and pitfalls in introducing climate change into the medical curricula recognized the importance of climate and health literacy on all levels, also those with the power to make curricular decisions (54).

We acknowledge that this article is a narrative report and should not be seen as a systematic review covering all relevant studies in the field. The selection and interpretation of studies rely on our perspective, potentially introducing bias, neither was the study quality assessed systematically. Despite these limitations, our study can provide an overview of the emerging fields of planetary health and identify some challenges and suggested solutions.

## 5 Conclusion

In conclusion, the roots of planetary health, the enormous health risks at stake and nature of the subject requires medical training to adopt a true inter/trans-disciplinary approach to succeed. It might not be expected for all students to become planetary health experts, but all need a general understanding of the most important aspects and values.

## Author contributions

EM: Conceptualization, Formal analysis, Funding acquisition, Investigation, Methodology, Project administration, Resources, Supervision, Validation, Visualization, Writing – original draft, Writing – review & editing. AO: Conceptualization, Data curation, Formal analysis, Funding acquisition, Investigation, Methodology, Project administration, Resources, Supervision, Validation, Visualization, Writing – original draft, Writing – review & editing.

## References

[1] Whitmee S Haines A Beyrer C Boltz F Capon AG Ferreira B. Safeguarding human health in the Anthropocene epoch: report of the Rockefeller Foundation–Lancet commission on planetary health. Lancet. (2015) 386:1973–2028. doi: 10.1016/S0140-6736(15)60901-1, PMID: 26188744

[2] Carson R. Silent spring In: Thinking about the environment. London: Routledge (2015)

[3] Sargent F 2nd. Man-environment--problems for public health. Am J Public Health. (1972) 62:628–33. doi: 10.2105/AJPH.62.5.628, PMID: 5024286 PMC1530249

[4] Tsaregorodtsev GI. Dialectics of interaction of economic and humanistic approaches. Sov Stud Philos. (1974) 13:100–6. doi: 10.2753/RSP1061-1967130203100

[5] Prescott SL Logan AC. Planetary health: from the wellspring of holistic medicine to personal and public health imperative. Explore. (2019) 15:98–106. doi: 10.1016/j.explore.2018.09.002, PMID: 30316687

[6] Booth AL. Environmental consciousness-native American worldviews and sustainable natural resource management: An annotated bibliography. Monticello, IL: Council of Planning Librarians (1988).

[7] Alvord L Van Pelt EC. The scalpel and the silver bear: The first Navajo woman surgeon combines western medicine and traditional healing. New York: Bantam (2000).

[8] The Lancet. A commission on climate change. Amsterdam: Elsevier (2009).

[9] Salas RN. The climate crisis and clinical practice. N Engl J Med. (2020) 382:589–91. doi: 10.1056/NEJMp2000331 32053295

[10] Cerceo E Vasan N. Creating environmental health leaders when educators are learning too. J Med Educat Curri Develop. (2023) 10:23821205231219162. doi: 10.1177/23821205231219162, PMID: 38130832 PMC10734366

[11] El Omrani O Dafallah A Castillo BP Amaro BQRC Taneja S Marouane Amzil M. Envisioning planetary health in every medical curriculum: an international medical student organization’s perspective. Med Teach. (2020) 42:1107–11. doi: 10.1080/0142159X.2020.1796949 32757869

[12] Bevan J Blyth R Russell B Holtgrewe L Cheung AHC Austin I. Planetary health and sustainability teaching in UK medical education: a review of medical school curricula. Med Teach. (2023) 45:623–32. doi: 10.1080/0142159X.2022.2152190, PMID: 36503358

[13] Tun SYM. Fulfilling a new obligation: teaching and learning of sustainable healthcare in the medical education curriculum. Med Teach. (2019) 41:1168–77. doi: 10.1080/0142159X.2019.1623870, PMID: 31237167

[14] Blanchard OA Greenwald LM Sheffield PE. The climate Change conversation: understanding Nationwide medical education efforts. Yale J Biol Med. (2023) 96:171–84. doi: 10.59249/PYIW9718, PMID: 37396984 PMC10303250

[15] Siddaway AP Wood AM Hedges LV. How to do a systematic review: a best practice guide for conducting and reporting narrative reviews, meta-analyses and meta-syntheses. Annu Rev Psychol. (2019) 70:747–70. doi: 10.1146/annurev-psych-010418-102803 30089228

[16] MacKenzie-Shalders K Zadow G Hensley-Hackett K Marko S McLean M. Rapid review: guides and frameworks to inform planetary health education for health professions. Health Promot J Austr. (2023). doi: 10.1002/hpja.819, PMID: 37866347

[17] Hampshire K Islam N Kissel B Chase H Gundling K. The planetary health report card: a student-led initiative to inspire planetary health in medical schools. Lancet Planet Health. (2022) 6:e449–54. doi: 10.1016/S2542-5196(22)00045-6 35461572

[18] Walpole SC Barna S Richardson J Rother H-A. Sustainable healthcare education: integrating planetary health into clinical education. Lancet Planetary Health. (2019) 3:e6–7. doi: 10.1016/S2542-5196(18)30246-8, PMID: 30654868

[19] Shaw E Walpole S McLean M Alvarez-Nieto C Barna S Bazin K. AMEE consensus statement: planetary health and education for sustainable healthcare. Med Teach. (2021) 43:272–86. doi: 10.1080/0142159X.2020.1860207, PMID: 33602043

[20] Baumgartner Jill. (2021). Planetary health: Protecting nature to protect ourselves. Oxford: Oxford University Press, 50, 697–698.

[21] Capetola T Noy S Patrick R. Planetary health pedagogy: preparing health promoters for 21st-century environmental challenges. Health Promot J Austr. (2022) 33:17–21. doi: 10.1002/hpja.641, PMID: 35866385 PMC9804771

[22] Redvers N Faerron Guzmán CA Parkes MW. Towards an educational praxis for planetary health: a call for transformative, inclusive, and integrative approaches for learning and relearning in the Anthropocene. Lancet Planet Health. (2023) 7:e77–85. doi: 10.1016/S2542-5196(22)00332-1, PMID: 36608953

[23] Redvers N Schultz C Vera Prince M Cunningham M Jones R Blondin B. Indigenous perspectives on education for sustainable healthcare. Med Teach. (2020) 42:1085–90. doi: 10.1080/0142159X.2020.1791320, PMID: 32657230

[24] Guzmán CA Faerron AA Aguirre BA Barros E Bayles B Chimbari M. A framework to guide planetary health education. Lancet Planet Health. (2021) 5:e253–5. doi: 10.1016/S2542-5196(21)00110-8, PMID: 33894134

[25] Gruetzmacher K Karesh WB Amuasi JH Arshad A Farlow A Gabrysch S. The Berlin principles on one health – bridging global health and conservation. Sci Total Environ. (2021) 764:142919. doi: 10.1016/j.scitotenv.2020.142919, PMID: 33097250 PMC7550087

[26] de Paula N. Planetary health diplomacy and transdisciplinary research. Eur J Pub Health. (2021) 31:422. doi: 10.1093/eurpub/ckab164.422

[27] Mago A Dhali A Kumar H Maity R Kumar B. Planetary health and its relevance in the modern era: a topical review. Sage Open Med. (2024) 12:20503121241254231. doi: 10.1177/20503121241254231, PMID: 38774741 PMC11107315

[28] Prescott SL Logan AC. Larger than life: injecting Hope into the planetary health paradigm. Challenges. (2018) 9:13. doi: 10.3390/challe9010013

[29] Padha S Kumar R Dhar A Sharma P. Microplastic pollution in mountain terrains and foothills: a review on source, extraction, and distribution of microplastics in remote areas. Environ Res. (2022) 207:112232. doi: 10.1016/j.envres.2021.112232, PMID: 34687754

[30] IPCC. IPCC Climate Change 2014. 'Impacts, adaptation and vulnerability', Part A: global and sectoral aspects Contribution of working group II to the fifth assessment report of the intergovernmental Panel on Climate Change. Cambridge: Cambridge University Press (2014).

[31] Pörtner H-O Scholes RJ Almut Arneth DKA Barnes MT Burrows SED Duarte CM. Overcoming the coupled climate and biodiversity crises and their societal impacts. Science. (2023) 380:eabl4881. doi: 10.1126/science.abl4881, PMID: 37079687

[32] Rockström J Steffen W Noone K Persson Å Chapin FS Lambin E. Planetary boundaries exploring the safe operating space for humanity. Ecol Soc. (2009) 14

[33] Steffen W Richardson K Rockström J Cornell SE Fetzer I Bennett EM. Planetary boundaries: guiding human development on a changing planet. Science. (2015) 347:1259855. doi: 10.1126/science.1259855, PMID: 25592418

[34] McMichael AJ. Global warming, ecological disruption and human health: the penny drops. Med J Aust. (1991) 154:499–501. doi: 10.5694/j.1326-5377.1991.tb119437.x, PMID: 2017079

[35] Halonen JI Erhola M Furman E Haahtela T Jousilahti P Barouki R. A call for urgent action to safeguard our planet and our health in line with the Helsinki declaration. Environ Res. (2021) 193:110600. doi: 10.1016/j.envres.2020.110600, PMID: 33307082

[36] Rosenberg CE. The cholera years: The United States in 1832, 1849, and 1866. Chicago, IL: University of Chicago Press (2009).

[37] Rosner D Markowitz GE. Deadly dust: Silicosis and the on-going struggle to protect workers' health. Ann Arbor, MI: University of Michigan Press (2006).

[38] Hall W. Cigarette century: The rise, fall and deadly persistence of the product that defined America, vol. 16. London: BMJ Publishing Group Ltd (2007). 360 p.

[39] Dunk JH Jones DS Capon A Anderson WH. Human health on an ailing planet — historical perspectives on our future. N Engl J Med. (2019) 381:778–82. doi: 10.1056/NEJMms1907455, PMID: 31433929

[40] Campbell-Lendrum D Neville T Schweizer C Neira M. Climate change and health: three grand challenges. Nat Med. (2023) 29:1631–8. doi: 10.1038/s41591-023-02438-w, PMID: 37464036

[41] Bell R Khan M Romeo-Velilla M Stegeman I Godfrey A Taylor T. Ten lessons for good practice for the INHERIT triple win: health, equity, and environmental sustainability. Int J Environ Res Public Health. (2019) 16:4546. doi: 10.3390/ijerph16224546, PMID: 31744247 PMC6888316

[42] Deguen S Zmirou-Navier D. Social inequalities resulting from health risks related to ambient air quality--a European review. Eur J Pub Health. (2010) 20:27–35. doi: 10.1093/eurpub/ckp220, PMID: 20081212

[43] Kuczmarski TM Fox J Katznelson E Thakral D Aung K-K Moore E. Climatizing the internal medicine residency curriculum: a practical guide for integrating the topic of climate and health into resident education. J Clim Change Health. (2021) 4:100067. doi: 10.1016/j.joclim.2021.100067

[44] World Health Organization. Healthy environments for healthier populations: Why do they matter and what can we do? Geneva: World Health Organization (2019).

[45] Bline AP DeWitt JC Kwiatkowski CF Pelch KE Reade A Varshavsky JR. Public health risks of PFAS-related Immunotoxicity are real. Curr Environ Health Rep. (2024) 11:118–27. doi: 10.1007/s40572-024-00441-y, PMID: 38526771 PMC11081924

[46] McKinney A. A planetary health approach to study links between pollution and human health. Curr Pollut Rep. (2019) 5:394–406. doi: 10.1007/s40726-019-00131-6

[47] Walpole SC Mortimer F. Evaluation of a collaborative project to develop sustainable healthcare education in eight UK medical schools. Public Health. (2017) 150:134–48. doi: 10.1016/j.puhe.2017.05.014, PMID: 28686956

[48] Stenvinkel P. Biomimetics and planetary health – a review of human diseases in interaction with the environment and ecosystem. Dan Med J. (2023) 71:A08230539. 38235986

[49] Newell R. The climate-biodiversity-health nexus: a framework for integrated community sustainability planning in the Anthropocene. Front Clim. (2023) 5:1177025. doi: 10.3389/fclim.2023.1177025

[50] Simon J Parisi S Wabnitz K Simmenroth A Schwienhorst-Stich EM. Ten characteristics of high-quality planetary health education-results from a qualitative study with educators, students as educators and study deans at medical schools in Germany. Front Public Health. (2023) 11:1143751. doi: 10.3389/fpubh.2023.1143751, PMID: 37181714 PMC10166869

[51] Cantor A DeLauer V Martin D Rogan J. Training interdisciplinary “wicked problem” solvers: applying lessons from HERO in community-based research experiences for undergraduates. J Geogr High Educ. (2015) 39:407–19. doi: 10.1080/03098265.2015.1048508

[52] Holm P Goodsite ME Cloetingh S Agnoletti M Moldan B Lang DJ. Collaboration between the natural, social and human sciences in global change research. Environ Sci Pol. (2013) 28:25–35. doi: 10.1016/j.envsci.2012.11.010

[53] Lyall C Bruce A Tait J Meagher L. Interdisciplinary research journeys: Practical strategies for capturing creativity. London: Bloomsbury Academic (2011).

[54] Greenwald L Blanchard O Hayden C Sheffield P. Climate and health education: a critical review at one medical school. Front Public Health. (2022) 10:1092359. doi: 10.3389/fpubh.2022.1092359 36711353 PMC9878448

